# Comparability of Daily-Life Walking Speed Measured by Smartphone GPS and Ankle-Band Accelerometer: Cross-Sectional Study

**DOI:** 10.2196/73722

**Published:** 2025-08-18

**Authors:** Hisashi Kawai, Keigo Imamura, Rui Gong, Manami Ejiri, Shuichi Obuchi

**Affiliations:** 1 Research Team for Human Care, Tokyo Metropolitan Institute for Geriatrics and Gerontology, 35-2 , Sakae-cho, Itabashi-ku, Tokyo, 1730015, Japan, 81 339643241 ext 4243, 81 339641844

**Keywords:** daily-life walking speed, accelerometer, GPS, smartphone, wearable health technology, ankle-band accelerometer, walking speed assessment

## Abstract

**Background:**

Daily-life walking speed (DWS), which is a critical health indicator in older adults, can be measured using smartphone GPS technology. Although this method is becoming more widely accessible**,** it is restricted to outdoor walking. In contrast, accelerometers can capture walking speed indoors; however**,** there is limited information on the comparability of DWS data between these two techniques.

**Objective:**

In this study, we aimed to investigate the agreement and systematic error between DWS measured using the built-in GPS of a smartphone and an ankle-band accelerometer.

**Methods:**

Participants were recruited from a previously selected cohort of community-dwelling older adults. Their DWS was assessed using both a smartphone app and ankle band accelerometer. Data from the two devices were matched based on simultaneous recordings, and agreement between the walking speeds was evaluated using the intraclass correlation coefficient (ICC) and a Bland–Altman plot.

**Results:**

A total of 99 participants (38 men, 61 women; mean [SD] age 71.5 [4.9] y) were included, yielding 3652 paired data points. The mean (SD) DWS as measured by GPS was 1.30 (0.19) m/s, and this was significantly higher than the value of 1.12 (0.23) m/s measured by the accelerometer (*P*<.001). The ICC(2, 1) (95% CI) value was 0.523 (−0.022 to 0.765), and the ICC(2, k) value was 0.687 (−0.045 to 0.867). The Bland–Altman plot revealed a fixed error of 0.18 m/s with 95% limits of agreement ranging from −0.49 m/s to 0.13 m/s in the GPS-measured walking speed compared to that measured by the ankle-band accelerometer.

**Conclusions:**

The GPS app consistently recorded a faster walking speed than the ankle-band accelerometer. The agreement between the measurements of the two devices was poor. The data suggest that a correction is necessary when comparing the DWS between these two devices.

## Introduction

Walking speed is a crucial indicator of various health outcomes in older adults, including instrumental activities of daily living disability [1], hospitalization [2], and mortality [3]. Previous studies have measured walking speed in controlled laboratory settings, typically on a walkway. However, recent advancements in wearable sensor technology have facilitated the measurement of daily-life walking speed (DWS) [4-9]. Unlike laboratory-based walking assessments, DWS captured by wearable sensors reflects natural, unintentional walking behavior, which makes it a valuable tool for daily health monitoring. A meta-analysis indicated that the use of activity trackers increased the number of steps taken per day and the moderate-to-vigorous physical activity duration [10]. Their results demonstrated that providing participants with daily feedback on their walking may encourage behavioral changes that promote better health.

We previously reported that the test-retest reliability of 1 week of DWS measured via smartphone GPS was adequate [7], that 1 month of DWS data was significantly associated with frailty [6,11], and that DWS distribution over a certain period followed a unimodal normal distribution [12]. These observations highlighted the potential utility of GPS in preventing frailty. In Japan, the smartphone ownership rate among people aged ≥70 years is 60.6% [13]. Therefore, GPS-based DWS measurement has the potential for widespread adoption. However, a key limitation of GPS technology is that it only captures outdoor walking data. As frailty progresses and they spend more time indoors, assessing indoor walking speed will become increasingly important.

Accelerometers provide an alternate method for measuring indoor walking speed. Previous studies have used wrist- [4,5,14], waist- [15], and ankle-band accelerometers [16,17] for this purpose. Ankle-band accelerometers directly measure foot movements during walking and are more accurate than waist-band accelerometers for classifying walking speeds [16]. The DWS distributions measured by accelerometers have exhibited a bimodal pattern that allows for the differentiation between indoor and outdoor walking speeds [15]. In contrast, DWS distribution captured by smartphone GPS typically yields a unimodal distribution. These output differences demonstrate the need for comparative studies to compare the DWS measured by each device. Despite the importance of such comparisons, no such research exists.

This study aimed to investigate the agreement and systematic error between DWS measured using the built-in GPS of a smartphone and an ankle-band accelerometer, which is considered to be the most accurate accelerator-based method for measuring walking speed. We also examined the comparability of DWS measurements obtained using these two devices.

## Methods

### Participants

Participants were recruited from the “Otassha Study 2011” cohort during the 2022 survey as part of a life-log monitoring project using wearable sensors [18,19]. This cohort study recruited residents of nine areas within Itabashi Ward, Tokyo, and conducted annual comprehensive health assessments. A total of 623 individuals participated in the 2022 survey, of whom 276 consented to 1 year of life-log monitoring.

This life-log monitoring project, conducted as a research project by the Tokyo Metropolitan Government, is officially known as “Smart Watch Innovation for Next Geriatrics and Gerontology (SWING-Japan).” In this project, the walking, sleep, and physical activity of participants were continuously monitored over 1 year using a smartwatch, an ankle-band accelerometer, and a smartphone application. Participants were instructed to wear these devices consistently during their daily activities.

In the follow-up cohort survey conducted 1 year later, participants in the present study consented to use data collected via the smartphone app and ankle-band accelerometer. 

### Ethical Considerations

The purpose and procedures of the study were explained to the participants both orally and in writing, and written informed consent was obtained from all individuals. This study was approved by the ethics committee of the Tokyo Metropolitan Institute for Geriatrics and Gerontology (Approval number: R22-094). The data were de-identified. No compensation was provided to the participants.

### DWS Measurement 

Walking speed, step length, and cadence during daily life were measured using a smartphone app (Kenko Choujyu App, TMIG, Tokyo, Japan) and an ankle-band accelerometer (WALK X, ACOS Co., Ltd., Nagano, Japan). The participants used their personal smartphones in this study. The app determined the location using the built-in GPS of the smartphone. Walking activity was detected using a built-in step counter app programming interface. The app began measuring walking speed when stable outdoor walking was detected and continued recording until the end of the session. The GPS measured the walking status and position every second, and the walking speed was calculated from the distance and time during stable walking. The number of steps taken during stable walking was obtained from the step counter, and cadence and step length were also calculated. The test-retest reliability and validity of the walking speed measurements obtained via this app were verified in a previous study [7]. Although a variety of communication environments such as Wifi, 5G, and 4G were available, the smartphone app only used mobile data when uploading the required information, and there was no impact on the measured values.

An ankle-band accelerometer was securely attached to the upper lateral malleolus of each participant. This device used a patented algorithm (Patent number: 6843332) to calculate the walking speed, step length, and cadence for each walking cycle based on the height of the participant and signals from a three-axis acceleration sensor. The accelerometer was connected to a smartphone app via Bluetooth, and the data were transferred to a cloud-based storage with timestamps that used mobile data from the smartphone. The accelerometer calculated walking speed for each walking cycle, and the average value for 1 min was recorded. The 1-minute average values were transmitted and maintained in cloud-based storage during long-term monitoring. The validity of this ankle-band accelerometer, compared to stopwatch measurements, was confirmed in a previous study [17]. Daily walking data from the smartphone app and the ankle-band accelerometer were stored in the cloud, along with corresponding timestamps.

### Data Matching

Daily-life walking data that had been recorded between November 1, 2022, and June 1, 2023 were obtained from the cloud for this study. The smartphone app data were converted to minute-by-minute datasets by truncating the seconds from each timestamp. These adjusted data were then synchronized with the corresponding minute-by-minute data measured using the ankle-band accelerometer.

### Statistical Analysis

Participant characteristics such as age, height, weight, normal walking speed in a laboratory environment, Mini-Mental State Examination-Japanese (MMSE-J) score, and prevalence of hypertension, stroke, heart disease, and diabetes were summarized as means and SD. These parameters were obtained from the cohort survey.

Normal walking speed was assessed by measuring the time required to walk the middle 5 m of an 11 m walkway (3 m acceleration + middle 5 m + 3 m deceleration). The measurement was performed twice, and the better of the two results was used. The MMSE-J was administered by a trained examiner, and the presence of chronic diseases was determined through nurse-conducted interviews.

The differences in the daily-life walking parameters measured by the two devices were assessed using paired *t* tests. Agreement between devices was measured using intraclass correlation coefficients (ICCs) ICC(2,1) and ICC(2,k). As the DWS is often represented by the average value for a week or month [7,11], we also examined the ICC(2, k) to determine the reliability across multiple measurements. Based on the 95% CI of the ICC estimate, we assessed values <0.5, 0.5‐0.75, 0.75‐0.9, and >0.9 as indicative of poor, moderate, good, and excellent reliability, respectively [20]. Distributional patterns were visualized using violin plots, and additional examinations that were performed after walking bouts <100 m were excluded. The DWS was assessed for systematic errors using Bland–Altman Plots. The mean percentage error (MPE) and mean absolute percentage error (MAPE) for DWS were also calculated as follows using the formulae from previous research [21]:


$$MPE=\frac{\left(acclerometerDWS\right)-(GPSDWS)}{accelerometerDWS}\times 100$$

$$MAPE=\frac{\left(acclerometerDWS\right)-(GPSDWS)}{accelerometerDWS}\times 100$$

All statistical analyses were conducted using SPSS Statistics 27 (IBM Japan, Ltd., Tokyo) and R version 4.4.1. We did not use generative AI to produce any portion of this work.

## Results

**Figure 1.**
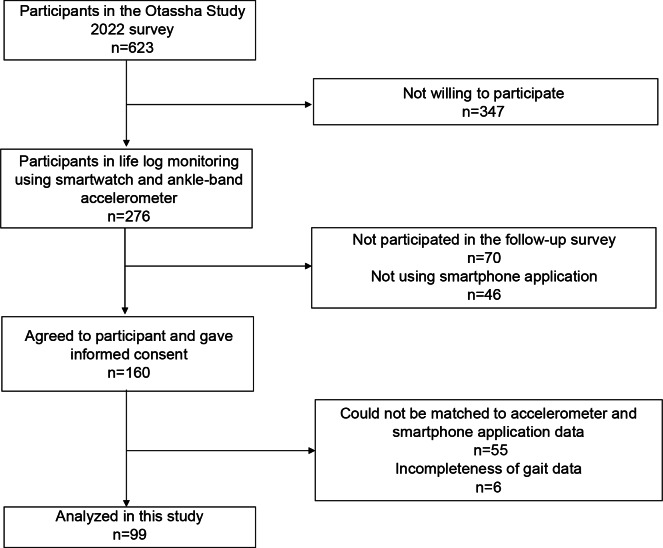
Flowchart of the study participants.

A total of 99 participants, whose walking data matched those from the smartphone app and ankle-band accelerometer, were included in the final analysis (Figure 1). The study included 99 participants (38 men and 61 women), with a mean (SD) age of 71.5 (4.9) years. The mean normal walking speed was 1.5 (0.3) m/s, and the mean MMSE-J score was 28.8 (1.5). The prevalence rates of hypertension, stroke, heart disease, and diabetes were 41.4%, 7.1%, 21.2%, and 10.1%, respectively (Table 1).

**Table 1.** Participant characteristics.

Characteristics	Men (n=38)	Women (n=61)	Overall (n=99)
Age (years), mean (SD)	71.5 (4.9)	71.5 (5.0)	71.5 (4.9)
Height (cm), mean (SD)	167.7 (5.4)	153.4 (5.6)	158.9 (8.9)
Weight (kg), mean (SD)	66.9 (10.4)	53.3 (9.6)	58.5 (11.9)
Normal walking speed (m/s), mean (SD)	1.5 (0.3)	1.5 (0.2)	1.5 (0.3)
MMSE-J^a^ score, mean (SD)	28.3 (2.0)	29 (1.1)	28.8 (1.5)
Hypertension, n (%)	18 (47.4)	23 (37.7)	41 (41.4)
Stroke, n (%)	3 (7.9)	4 (6.6)	7 (7.1)
Heart disease, n (%)	9 (23.7)	12 (19.7)	21 (21.2)
Diabetes, n (%)	7 (18.4)	3 (4.9)	10 (10.1)

a MMSE-J: Mini-Mental State Examination-Japanese.

The average (SD) measurement period was 121.1 (68.2) days. The smartphone app recorded 6527 minute-by-minute walking data points over the measurement period; however, only 3652 data points could be matched with the accelerometer data. The mean (SD) DWS measured by GPS was 1.30 (0.19) m/s, which was significantly higher than the value measured by the accelerometer of 1.12 (0.23) m/s (*P*<.001; Table 2). The step length and cadence recorded by the GPS were also significantly greater than those measured by the accelerometer (*P*<.001). The ICC(2,1) [95% CI] values for DWS, step length, and cadence measured using the two devices were 0.523 [−0.022 to 0.765], 0.297 [−0.068 to 0.557], and 0.440 [0.385 to 0.490], respectively, and the ICC(2,k) [95% CI] values were 0.687 [−0.045 to 0.867], 0.458 [−0.147 to 0.715], and 0.611 [0.556 to 0.657], respectively (Table 2).

**Table 2.** Comparisons of daily-life walking speed, step length, and cadence measured by the GPS and ankle-band accelerometer.

Variables	GPS			ACC^a^		*P* value	ICC^b^ (2,1)	95% CI	ICC (2,k)	95% CI
	Observation	Mean (SD)		Mean (SD)						
Walking speed (m/s)	3652	1.30 (0.19)		1.12 (0.23)		<.001	0.523	−0.022 to 0.765	0.687	−0.045 to 0.867
Step length (m)	3652	0.67 (0.08)		0.57 (0.09)		<.001	0.297	−0.068 to 0.557	0.458	−0.147 to 0.715
Cadence (step/min)	3652	116.72 (13.13)		111.80 (21.09)		<.001	0.440	0.385 to 0.490	0.611	0.556 to 0.657

a ACC: ankle-band accelerometer.

b ICC: intra-class correlation coefficient.

**Figure 2.**
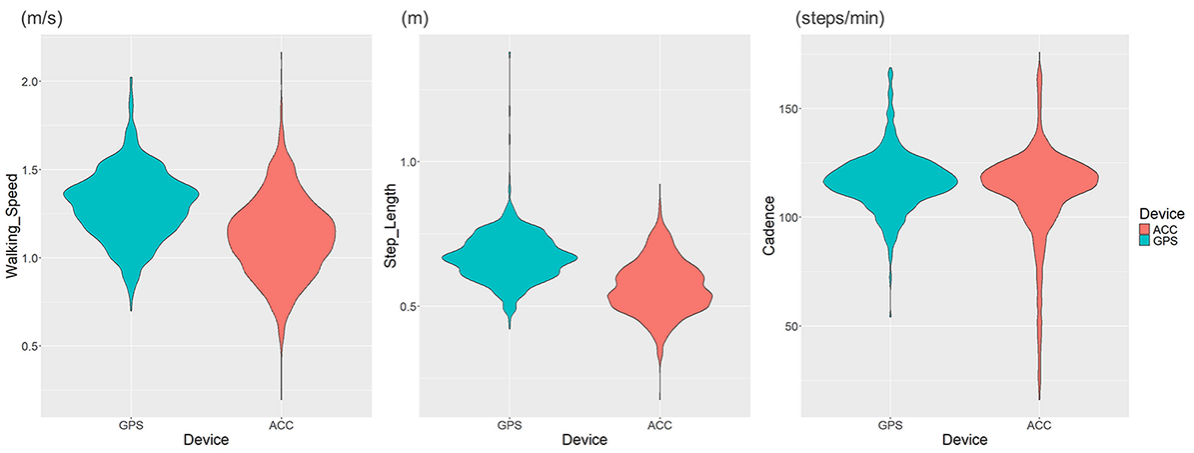
Violin plots of daily walking speed, step length, and cadence measured using GPS and an ankle-band accelerometer (ACC) (observations=3652).

Violin plots of daily-life walking parameters measured using the two devices demonstrated that GPS-measured walking speeds were consistently higher than those recorded using the accelerometer (Figure 2). The discrepancy in the distribution was more pronounced for step length than for cadence. These trends persisted even when walking bouts of <100 m were excluded (Supplementary Figure 1 in Multimedia Appendix 1).

**Figure 3.**
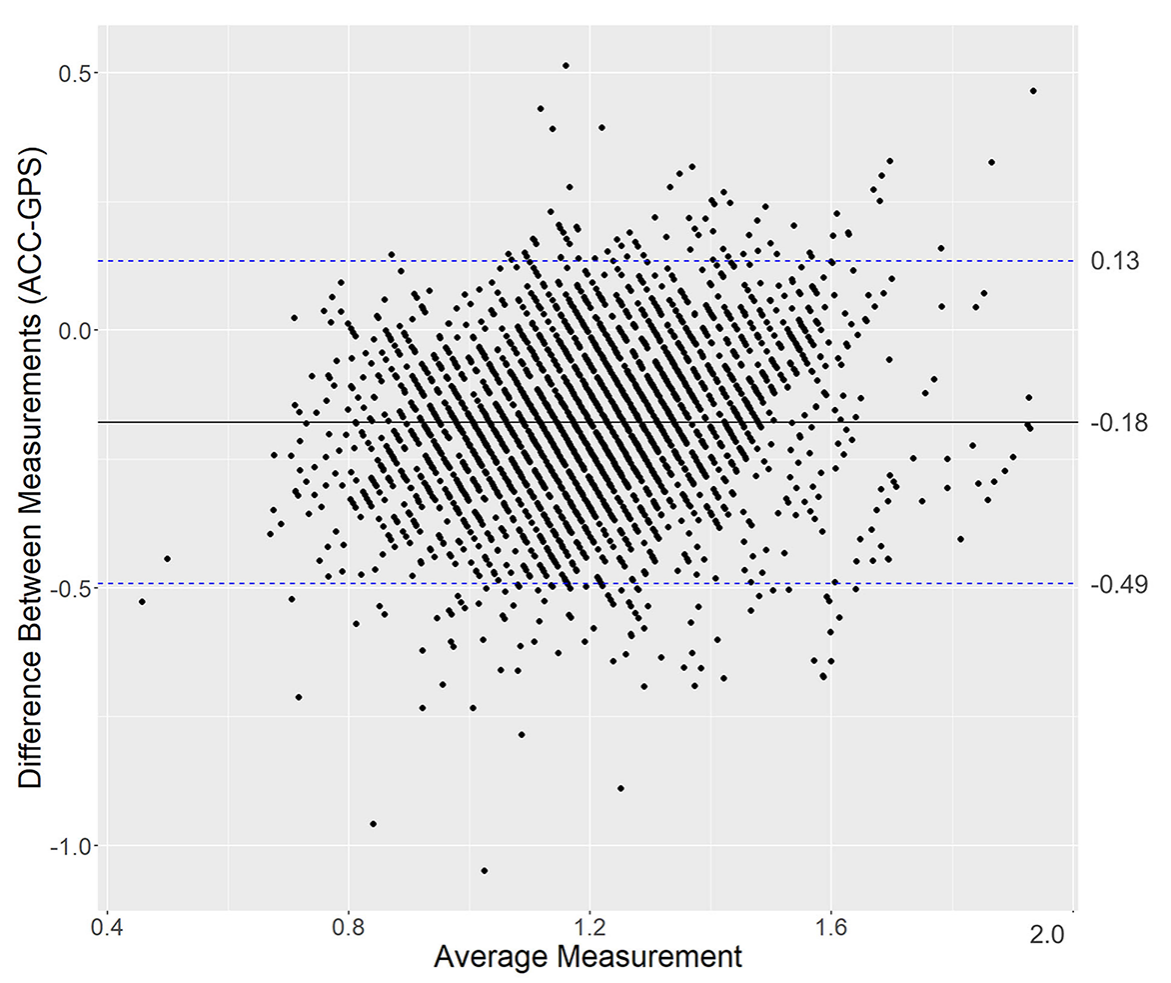
Bland-Altman plots of comparing daily walking speed measurements obtained from GPS and an ankle-band accelerometer (ACC) (Observations=3652). Dotted lines: 95% limit of agreement.

A Bland–Altman plot, which was used to depict the differences between DWS measurements obtained by the accelerometer and GPS on the vertical axis, revealed a negatively skewed distribution (Figure 3). The mean difference in DWS between the two devices was −0.18 m/s, with 95% limits of agreement ranging from −0.49 m/s to 0.13 m/s. The MPE and MAPE for DWS (SD) were −18.5% (19.2) and 19.9% (17.8), respectively.

## Discussion

### Principal Results

This study compared DWS measurements of community-dwelling older adults obtained from a smartphone GPS and an ankle-band accelerometer. The normal walking speed measured in the cohort survey was 1.5 m/s, which was faster than the 1.25‐1.29 m/s reported in the Japanese integral cohort study [22]. Additionally, the prevalence of hypertension, stroke, heart disease, and diabetes among participants tended to be lower in the present study. These findings suggest that individuals who participated in long-term measurements using wearable sensors were healthier than the representative cohort of community-dwelling older people in Japan.

Although we included data spanning up to 7 months in the analyses, the mean number of paired data points per participant was small (36.9). Some participants had only a few paired data points despite long measurement periods, indicating that the thresholds for detecting walking differed between the two devices.

The agreement between the two devices was poor, except for the ICC(2,k) of cadence, and not compatible. The range of 95% limits of agreement for DWS was wide (from −0.49 to 0.13 m/s). Although the 95% minimum detection change for walking speed is approximately 0.1‐0.2 m/s [23], in the present study, the negative side, in particular, was significantly larger, suggesting that the two measurements were not comparable. The MPE and MAPE suggested that the two measurements exhibited unacceptable percentages of error. The violin plots showed that the GPS had a narrower measurement range than the accelerometer, which shifted the distribution toward a faster walking speed. This observation suggested that the accelerometer was more sensitive to slower walking speeds.

### Comparison With Prior Work

A previous study reported that an ankle-band accelerometer had greater classification accuracy for walking speed than accelerometers attached to other body locations [16]. In the present study, the ankle-band accelerometer might have provided a more accurate measurement of slower walking speeds over short outdoor walking bouts. A previous study on DWS that included indoor walking showed that short walking bouts of 5‐10 steps (equivalent to 3‐7 m) accounted for more than 40% of all walking [24]. However, in the present study, which was limited to outdoor walking, the accelerometer detected walking for distances <100 m in 47.3% of cases, whereas only 9.4% were detected by the GPS. However, excluding walking bouts <100 m did not alter the overall distribution pattern, which indicated that the discrepancy between devices was not solely attributable to short-duration walks. Walking distances of >500 m accounted for 17.7% of the total GPS-detected walking bouts, but only 1.1% of those were detected by the ankle-band accelerator. This information suggested that the criteria for determining stable walking interruptions differed between the two devices. These results indicate that the GPS-based tracking does not detect short walking bouts as effectively as the ankle-band accelerator, but is more likely to capture longer walking bouts. However, since previous studies have demonstrated the reliability and validity of GPS-based measurements [7,11,12], this characteristic does not indicate that GPS is inherently less accurate in measuring DWS.

### Limitations

A key limitation of this study was that it used a single accelerometer and GPS product. Therefore, the extent to which these findings apply to other wearable devices remains uncertain. Nevertheless, the results reflected the high classification accuracy of the ankle-band accelerometer in detecting walking speeds and they may be generalizable to some extent. As ankle edema was not examined in this study, its effect on the accuracy of ankle-band accelerometers is unknown. Additionally, the degree of agreement between devices might have varied among older adults with frailty or specific medical conditions [25]. This study also analyzed DWS exclusively during outdoor walking. Future research should examine the relationship between indoor and outdoor DWS.

Potential participants for this study were 230 smartphone users who participated in the life-log monitoring project; however, 70 participants did not participate in the 1-year follow-up survey or did not consent to this study. Additionally, 61 participants who could not be matched with data dropped out, resulting in a high dropout rate of 57.0%. Approximately 30% of participants stopped life-log monitoring within 1 year. Moreover, maintaining the long-term wearing rate of wearable devices among older adults is challenging.

### Conclusions

This study compared DWS measurements between a smartphone GPS and an ankle-band accelerometer in community-dwelling older adults. The GPS consistently recorded faster walking speeds than the ankle-band accelerometer, with a fixed unacceptable error of 0.18 m/s. Correction factors should be applied when comparing data between these two devices. Further research is necessary to investigate the comparability of DWS measurements across different populations and walking environments.

## Supplementary material

10.2196/73722 Multimedia Appendix 1 Violin plots of daily walking speed, step length, and cadence measured by GPS and an ankle-band accelerometer (ACC) for walking distances greater than 100 m (Observations = 1,921).

## Abbreviations

DWS: daily-life walking speed
ICC: intraclass correlation coefficient
MAPE: mean absolute percentage error
MPE: mean percentage error
