# Supplementary figures and images for: Comparability of Daily-Life Walking Speed Measured by Smartphone GPS and Ankle-Band Accelerometer: Cross-Sectional Study
Source: JMIR Form Res. 2025 Aug 18;9:e73722. doi: 10.2196/73722 (PMC12361537; doi:10.2196/73722)

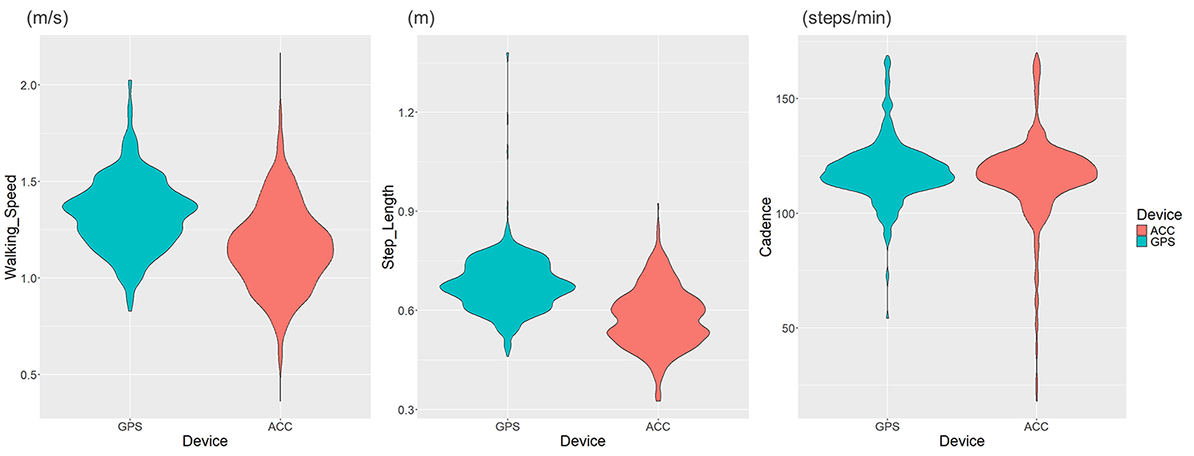

Supplement: Multimedia Appendix 1 [file formative-v9-e73722-s001.png]
